# Does Smoke from Biomass Fuel Contribute to Anemia in Pregnant Women in Nagpur, India? A Cross-Sectional Study

**DOI:** 10.1371/journal.pone.0127890

**Published:** 2015-05-29

**Authors:** Charlotte M. Page, Archana Patel, Patricia L. Hibberd

**Affiliations:** 1 Harvard Medical School, Boston, Massachusetts, United States of America; 2 Lata Medical Research Foundation, Nagpur, Maharashtra, India; 3 Division of Global Health, Massachusetts General Hospital for Children, Boston, Massachusetts, United States of America; University of Rochester Medical Center, UNITED STATES

## Abstract

**Background:**

Anemia affects upwards of 50% of pregnant women in developing countries and is associated with adverse outcomes for mother and child. We hypothesized that exposure to smoke from biomass fuel – which is widely used for household energy needs in resource-limited settings – could exacerbate anemia in pregnancy, possibly as a result of systemic inflammation.

**Objective:**

To evaluate whether exposure to smoke from biomass fuel (wood, straw, crop residues, or dung) as opposed to clean fuel (electricity, liquefied petroleum gas, natural gas, or biogas) is an independent risk factor for anemia in pregnancy, classified by severity.

**Methods:**

A secondary analysis was performed using data collected from a rural pregnancy cohort (N = 12,782) in Nagpur, India in 2011-2013 as part of the NIH-funded Maternal and Newborn Health Registry Study. Multinomial logistic regression was used to estimate the effect of biomass fuel vs. clean fuel use on anemia in pregnancy, controlling for maternal age, body mass index, education level, exposure to household tobacco smoke, parity, trimester when hemoglobin was measured, and receipt of prenatal iron and folate supplements.

**Results:**

The prevalence of any anemia (hemoglobin < 11 g/dl) was 93% in biomass fuel users and 88% in clean fuel users. Moderate-to-severe anemia (hemoglobin < 10 g/dl) occurred in 53% and 40% of the women, respectively. Multinomial logistic regression showed higher relative risks of mild anemia in pregnancy (hemoglobin 10-11 g/dl; RRR = 1.38, 95% CI = 1.19-1.61) and of moderate-to-severe anemia in pregnancy (RRR = 1.79, 95% CI = 1.53-2.09) in biomass fuel vs. clean fuel users, after adjusting for covariates.

**Conclusion:**

In our study population, exposure to biomass smoke was associated with higher risks of mild and moderate-to-severe anemia in pregnancy, independent of covariates.

**Trial Registration:**

ClinicalTrials.gov NCT 01073475

## Introduction

Anemia in pregnancy is a major public health problem: it is a common phenomenon, especially in resource-poor settings, and is associated with adverse outcomes for mother and child. The World Health Organization (WHO) estimates that 23% of pregnant women in industrialized countries and 52% of pregnant women in non-industrialized countries are anemic [1]. The prevalence of anemia in pregnancy in India is one of the highest in the world, with estimates consistently above 70% and some as high as 96% [2–4]. Anemic pregnant women are at higher risk of mortality due to ante- or postpartum hemorrhage and sepsis. According to the WHO, 64.4% of maternal deaths in India during 1992–1994 were associated with anemia [5].

In addition, the babies of anemic mothers are at increased risk of adverse outcomes such as stillbirth, premature delivery, low birth weight, and early neonatal mortality [1]. A cohort study in Pakistan found that fetuses of anemic mothers had a 3.7-fold greater risk of intrauterine death than non-anemic mothers [6].

Pregnancy itself is a cause of anemia: the blood volume expands by about 50% while the total red blood cell mass expands by only about 25%, resulting in so-called dilutional or physiological anemia of pregnancy [7]. This physiological anemia can be compounded by nutritional problems, including iron, folic acid, and vitamin B_12_ deficiencies. Iron deficiency is recognized as the foremost of these acquired causes of anemia in pregnancy [5]. Many Indian women begin pregnancy with iron deficiency, and this deficiency is compounded as iron requirements increase during the course of pregnancy [8]. Risk factors for anemia in pregnancy identified in previous studies include high parity [9], low body mass index (BMI) [10–11], iron-poor diet [7], short inter-pregnancy interval [7], low education level [10], and parasite infection [12].

After iron-deficiency anemia, the second most common type of anemia is anemia of inflammation. Anemia of inflammation occurs in patients with acute or chronic immune activation associated, for example, with infection, cancer, or autoimmune disease [13]. We hypothesize that chronic exposure to smoke from biomass fuel could cause systemic inflammation and thereby compound the effects of physiological changes and iron deficiency on anemia in pregnancy.

Biomass fuels—wood, straw, crop residues, and dung—are widely used by households in resource-poor countries. In 2004, an estimated 2.4 billion people worldwide relied on biomass fuels as their main source of energy for cooking, heating, and lighting [14]. These fuels are typically burned in inefficient, unvented cookstoves in poorly ventilated areas of the home [15]. This practice results in high levels of household air pollution, which is the leading environmental cause of morbidity and mortality worldwide [16]. To date, biomass smoke has been linked to increased risks of acute lower respiratory infections in children, chronic obstructive pulmonary disease, tuberculosis, lung cancer, nasopharyngeal and laryngeal cancer, cardiovascular disease, cataracts, low birth weight, and stillbirth [14,16–18].

Exposure to biomass smoke is highest among women, who do most of the cooking, and young children, who tend to stay indoors with their mothers [15]. In the past decade, studies have shown an association between exposure to biomass smoke and anemia in children [15,19]; however, this association has not been studied in pregnant women. Therefore, we conducted a secondary analysis of cross-sectional data from a large pregnancy cohort in Nagpur, India. The objective of the study was to evaluate whether exposure to smoke from biomass fuel (wood, straw, crop residues, or dung) as opposed to clean fuel (electricity, liquefied petroleum gas, natural gas, or biogas) is an independent risk factor for anemia in pregnancy, over and above known risk factors for which data were available.

## Materials and Methods

### Study design and data collection

A cross-sectional secondary data analysis was conducted using data from the Maternal and Newborn Health (MNH) Registry Study. The MNH is an ongoing, prospective cohort study that enrolls rural pregnant women and records pregnancy outcomes up to six weeks post-partum. It is overseen by the Global Network for Women and Children’s Health Research of the *Eunice Kennedy Shriver* National Institute of Child Health and Human Development, United States of America. Data collection began in 2008 and is ongoing in 106 low-resource geographic areas, or clusters, in Asia, Africa, and Latin America. Pregnant women are eligible to participate if they are either permanent residents of the study cluster and/or deliver within the study cluster. Data are collected at three time-points: on enrollment (which may occur at any point during pregnancy but is usually as soon as a woman is discovered to be pregnant), within 48 hours of delivery, and six weeks after birth. The MNH aims to study all eligible, consenting pregnant women in each cluster [20].

The current study uses data from pregnant women living in the 20 clusters in Nagpur, India. Each cluster consists of a population of approximately 30,000 that live in the villages in the catchment area of a primary health center. Pregnant women in the villages are invited to participate in the study and are enrolled after they provide written informed consent. Data are collected by medical officers and auxiliary nurse-midwives in primary health centers and sub-centers, both of which are part of India’s public healthcare system.

### Anemia variables

Anemia status of pregnant women was based on hemoglobin concentration, in g/dL, recorded within two weeks of enrollment. This measurement was performed using a Sahli hemometer at an antenatal care clinic at the sub-center or primary health center where the woman was enrolled.

Definitions from the WHO were used to classify pregnant women based on hemoglobin (Hb) level as not anemic (Hb ≥ 11 g/dl), mildly anemic (10 g/dl ≤ Hb < 11 g/dl), moderately anemic (7 g/dl ≤ Hb < 10 g/dl), or severely anemic (Hb < 7 g/dl) [21]. Because the proportion of severely anemic women in the study population was very small (< 1%), we used a three-category response variable for anemia: not anemic, mildly anemic, and moderately-to-severely anemic.

### Fuel variable

Data on fuel type were surveyed six weeks after birth using the question, “What type of fuel does your household mainly use for cooking?” Study participants were asked this question for both their primary house and for their secondary house—defined as a house in which they resided for more than 30 days during their pregnancy—if applicable. The answer choices were electricity, liquefied petroleum gas (LPG), natural gas, biogas, kerosene, coal/lignite, charcoal, wood, straw/shrubs/grass, agricultural crop, animal dung, no food cooked in household, and other. In general, the auxiliary nurse-midwives completing the forms could corroborate participants’ responses based on their own knowledge, as community members, of participants’ cooking practices. We classified electricity, LPG, natural gas, and biogas as clean fuels and wood, straw/shrubs/grass, agricultural crop, and animal dung as biomass fuel. We excluded kerosene, charcoal, coal, and lignite because fewer than 5% of households in Nagpur use these fuels, and the type of pollution from these fuels differs from that from biomass fuel [22].

### Other predictor variables

The other predictor variables included in the study were those collected by the MNH that are associated with anemia in pregnancy in the literature: maternal age, BMI, education level, exposure to household tobacco smoke, parity, trimester when hemoglobin was measured, and receipt of prenatal iron and folate supplements. Exposure to household tobacco smoke was ascertained at the third MNH time-point, i.e. six weeks after delivery. Data on prenatal iron and folate supplements were obtained at the second time-point, i.e. within 48 hours of delivery. Data for all other variables were collected at enrollment.

Maternal age and BMI were treated as continuous variables, with BMI calculated from recorded data on height (in cm) and weight (in kg). The remaining predictor variables were categorical. Education was categorized as no formal schooling or either starting or completing primary school (kindergarten through grade four), secondary school (grades five through 12), or university/college. Parity excluded the current pregnancy and was categorized as zero, one, two, and three or more. Parity of three or more was combined into one category because less than 1% of women were of parity greater than three.

Exposure to household tobacco smoke was ascertained with the question, “How often does anyone smoke inside your house? Would you say daily, weekly, monthly, less than monthly, or never?” and was treated as a five-level categorical variable. Active smoking by women is incredibly rare in our study population, so it was not included in the study.

The trimester when hemoglobin was measured was estimated as the timespan between the reported date of last menstrual period and the date of study enrollment (1^st^ trimester = 0–97 days, 2^nd^ trimester = 98–188 days, 3^rd^ trimester = 189–280 days of pregnancy). As noted previously, the date of enrollment may not have coincided exactly with the date of hemoglobin measurement but was generally within two weeks.

Receipt of prenatal iron and folate supplements was a yes/no variable. In Nagpur, pregnant women are given tablets of iron combined with folate (100 mg of elemental iron with 0.5 mg of folate) to take daily beginning in the 2^nd^ trimester. Women who are not anemic are given a minimum of a 100 days’ supply. Women who are anemic are given tablets to take twice daily beginning in the 2^nd^ trimester and continuing through the postpartum period.

### Participants

**Fig 1:**
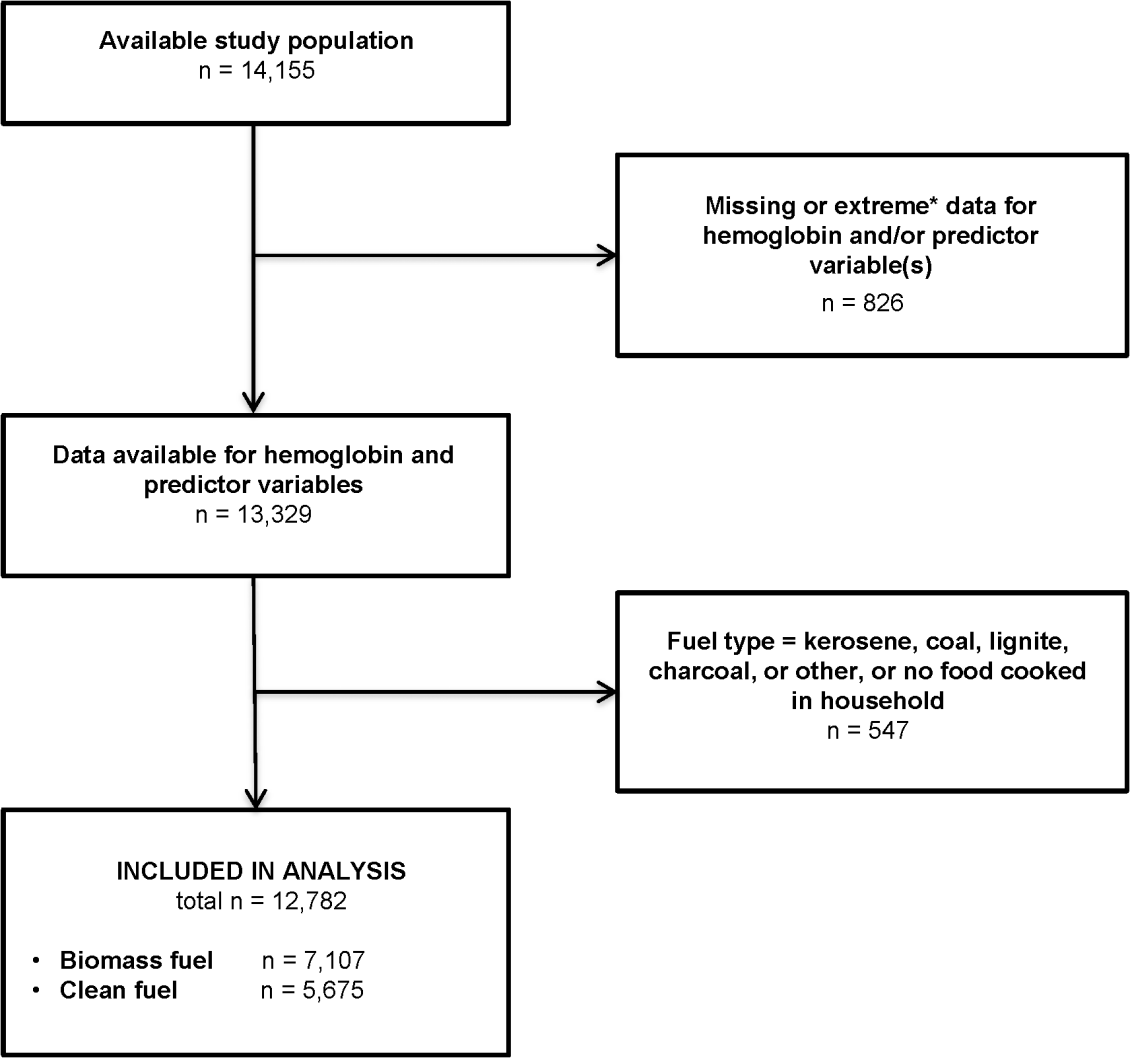
Flow diagram of study participants. Extreme values: for hemoglobin, hemoglobin ≤ 2 g/dl; for body mass index (BMI), height ≤ 120 cm or weight > 90 kg; for trimester, gestational age (as determined by last menstrual period) < 5 weeks or > 42 weeks.

The study population consisted of 14,155 women who completed the MNH study between May 2011 and June 2013 (Fig 1). Women with missing or extreme data for one or more study variable (n = 826) were excluded from the analysis. Extreme values were excluded because they were unlikely to be accurate. The following values were considered extreme: for hemoglobin, hemoglobin ≤ 2 g/dl; for BMI, height ≤ 120 cm or weight > 90 kg; and for trimester, gestational age (as determined by last menstrual period) < 5 weeks or > 42 weeks.

Of the remaining 13,329 women, 547 were excluded because their fuel type was neither clean nor biomass fuel (see above definitions). A total of 12,782 women were included in the analysis, comprising 7,107 biomass fuel users and 5,675 clean fuel users.

### Ethics Statement

The MNH study was approved by the Partners Human Research Committee (IRB# 2010P001511/MGH) and the Lata Medical Research Foundation’s Institutional Review Board. The MNH study was also registered at ClinicalTrials.gov (NCT 01073475). Participants provided written informed consent. The secondary data analysis was reviewed and deemed exempt by the Institutional Review Board at Harvard Medical School (IRB# 13–0463).

### Analysis

Bivariate analyses between anemia (stratified by severity) and each predictor variable were examined, in turn, using multinomial logistic (or logit) regression with a single predictor variable. For each predictor variable, this analysis yielded unadjusted relative risk ratios (RRRs) for the outcomes of mild anemia and moderate-to-severe anemia, compared to the baseline outcome of no anemia. For continuous predictor variables, namely age and BMI, the calculated RRRs are for a one-unit increase in the value of the variable. For the remaining, categorical predictor variables, an RRR was calculated for each category compared to a baseline category. A primer on interpretation of RRR is included as S1 Appendix, using an example provided in S1 Table [23].

All predictor variables found to be significantly associated with anemia (which happened to be all predictor variables) were included in a multivariable, multinomial logistic regression model. This model yielded RRRs adjusted to control for all predictor variables. Interactions between fuel type and other predictor variables were examined, but since none of them were statistically significant, no interaction terms were included in the model. Clustering at the level of the health centers was accounted for by including the health center as a categorical predictor variable in the regression model.

For all statistical tests, a two-sided p-value < 0.05 was considered significant. All analyses were performed using the statistical software program STATA/SE 12.1.

## Results

### Characteristics of participants

Table 1 presents the characteristics of the study participants, grouped by fuel type. 56% reported biofuel use while 44% reported clean fuel use, in their primary houses. 30.1% of study participants reported having a secondary house, 82.3% of which used the same fuel type (clean fuel vs. biofuel) as the primary house. Therefore, in total, only 5.3% of study participants spent more than 30 days during their pregnancy in a residence using a fuel type (clean fuel vs. biofuel) different than that in their primary house. Given the high concordance between fuel types in primary and secondary houses and the short duration of exposure, we included only fuel type for the primary house in our analysis.

**Table 1:** Characteristics of participants. Data expressed as mean (SD) or frequency (%).

Parameter	Category	Clean fuel users	Biomass fuel users	p-value
		n = 5,675	n = 7,107	
Age, in years	—	23.7 (2.9)	23.4 (2.9)	<0.001
BMI, in kg/m^2^	—	20.2 (2.8)	19.4 (2.6)	<0.001
Education	No formal schooling	95 (2%)	227 (3%)	
	Primary	811 (14%)	1,286 (18%)	
	Secondary	3,199 (56%)	4,451 (63%)	
	University/ college	1,570 (28%)	1,143 (16%)	<0.001
Exposure to household tobacco smoke	Never	3,487 (61%)	3,842 (54%)	
	Less than monthly	569 (10%)	495 (7.0%)	
	Monthly	280 (4.9%)	303 (4.3%)	
	Weekly	365 (6.4%)	551 (7.8%)	
	Daily	974 (17%)	1916 (27%)	<0.001
Parity, n	0	2,895 (51%)	3,301 (46%)	
	1	2,207 (39%)	2,830 (40%)	
	2	469 (8%)	742 (10%)	
	3+	104 (2%)	234 (3%)	<0.001
Trimester	1^st^	1,961 (35%)	2,584 (36%)	
	2^nd^	2,485 (44%)	3,049 (43%)	
	3^rd^	1,229 (22%)	1,474 (21%)	0.095
Prenatal iron and folate supplements	Yes	5,533 (98%)	6,915 (97%)	
	No	142 (2.5%)	192 (2.7%)	0.483
			

Trimester refers to the time when hemoglobin was measured.

Abbreviations: SD = standard deviation, BMI = body mass index.

On average, participants were young, the mean age being 23.7 years for clean fuel users and 23.4 years for biofuel users. The mean BMI was normal for both groups but higher in clean fuel users (20.2 kg/m^2^ vs. 19.4 kg/m^2^). The most common highest education level achieved for each group was secondary school (56% of clean fuel users and 63% of biomass fuel users), with a greater percentage of clean fuel users reaching university/college than biomass fuel users (28% compared to 16%). The majority of women did not have exposure to household tobacco smoke; this percentage was higher for clean fuel users (61%) than for biomass fuel users (54%). The two groups had similar distributions across pregnancy trimesters. For both groups, most women were either nulliparous or uniparous, and receipt of prenatal iron and folate supplements was the overwhelming norm.

### Bivariate analysis of risk factors

Overall, 90.5% of the women were anemic: 43.1% were mildly anemic, and 47.4% were moderately-to-severely anemic. The prevalence of any anemia was significantly higher in women living in households using biomass fuel (92.8%) than in women living in households using clean fuel (87.6%) (p-value < 0.0001). The difference in the prevalence of moderate-to-severe anemia in these two groups was more marked—53.1% compared to 40.3%, respectively (p-value < 0.0001).

Expressing these differences in prevalence in terms of relative risk, the unadjusted relative risk of moderate-to-severe anemia in pregnancy (relative to no anemia in pregnancy) was significantly greater among women in households using biomass fuel than among women in households using clean fuel (RRR = 2.26, 95% CI = 2.00–2.56). The unadjusted relative risk of mild anemia in pregnancy was also significantly greater among women in households using biomass fuel (RRR = 1.44, 95% CI = 1.27–1.64).

These RRRs are presented in Table 2, along with RRRs for other risk factors for anemia in pregnancy in our sample. In addition to fuel type, all other predictor variables included in the analysis were significantly associated with anemia. The relative risk of moderate-to-severe anemia in pregnancy decreased with increasing age, BMI, education, and trimester, whereas it increased with increasing parity. Women were more likely to be moderately-to-severely anemic if they were exposed to household tobacco smoke less than monthly compared to never. They were also more likely to be moderately-to-severely anemic if they did not receive prenatal iron and folate supplements (compared to receiving supplements).

**Table 2:** Bivariate analysis of risk factors for anemia in pregnant women in Nagpur, India, 2011–2013 (N = 12,782).

		Mild anemia		Moderate-to-severe anemia	
Risk factor	Category	RRR	95% CI	RRR	95% CI
Fuel type	Clean fuel	1.00*		1.00*	
	Biomass fuel	1.44	1.27–1.64^‡^	2.26	2.00–2.56^‡^
Age (years)	—	0.96	0.94–0.98^‡^	0.96	0.94–0.98^‡^
BMI (kg/m^2^)	—	0.92	0.90–0.94^‡^	0.83	0.81–0.85^‡^
Education	No formal schooling	1.00*		1.00*	
	Primary	0.78	0.46–1.33	0.56	0.33–0.95^‡^
	Secondary	0.73	0.43–1.22	0.50	0.30–0.83^‡^
	University/ college	0.52	0.31–0.87^‡^	0.26	0.16–0.44^‡^
Exposure to household tobacco smoke	Never	1.00*		1.00*	
	Less than monthly	1.29	1.09–1.52^‡^	1.68	1.43–1.98^‡^
	Monthly	1.24	0.96–1.59	1.24	0.96–1.59
	Weekly	1.46	1.06–2.01^‡^	1.31	0.95–1.81
	Daily	1.18	0.94–1.48	1.11	0.88–1.39
Parity (n)	0	1.00*		1.00*	
	1	1.18	1.04–1.35^‡^	1.31	1.15–1.50^‡^
	2	1.40	1.09–1.79^‡^	1.86	1.46–2.37^‡^
	3+	1.69	0.97–2.92	3.53	2.08–6.00^‡^
Trimester	1^st^	1.00*		1.00*	
	2^nd^	0.80	0.69–0.93^‡^	0.72	0.62–0.83^‡^
	3^rd^	0.81	0.68–0.96^‡^	0.50	0.42–0.59^‡^
Prenatal iron and folate supplements	Yes	1.00*		1.00*	
	No	1.41	0.88–2.23	1.74	1.10–2.75^‡^
				

Mild anemia and moderate-to-severe anemia are defined as 10 g/dl ≤ hemoglobin (Hb) < 11 g/dl and Hb < 10 g/dl, respectively.

* Baseline category.

^‡^ Differs significantly from 1 at the 95% confidence level.

Abbreviations: RRR = relative risk ratio, 95% CI = 95% confidence interval, BMI = body mass index.

### Multivariable analysis of risk factors

Controlling for the other predictors in the study—namely age, BMI, education, exposure to household tobacco smoke, parity, trimester, and prenatal iron and folate supplements—using multivariable, multinomial logistic regression reduced the RRRs for biofuel use, but they remained statistically significant. The adjusted relative risk of moderate-to-severe anemia in pregnancy was 1.79 (95% CI = 1.53–2.09) times greater among biomass fuel users than clean fuel users, and that for mild anemia in pregnancy was 1.38 (95% CI = 1.19–1.61) times greater among biomass fuel users than clean fuel users (Table 3).

**Table 3:** Multivariable analysis of risk factors for anemia in pregnant women in Nagpur, India, 2011–2013: multivariable, multinomial logistic regression model.

		Mild anemia		Moderate-to-severe anemia	
Risk factor	Category	RRR	95% CI	RRR	95% CI
Fuel type	Clean fuel	1.00*		1.00*	
	Biomass fuel	1.38	1.19–1.61^‡^	1.79	1.53–2.09^‡^
Age (years)	—	0.95	0.93–0.98^‡^	0.94	0.91–0.96^‡^
BMI (kg/m^2^)	—	0.92	0.90–0.94^‡^	0.83	0.81–0.86^‡^
Education	No formal schooling	1.00*		1.00*	
	Primary	0.81	0.47–1.41	0.55	0.32–0.95^‡^
	Secondary	0.78	0.46–1.32	0.50	0.29–0.85^‡^
	University/ college	0.60	0.35–1.02	0.30	0.17–0.51^‡^
Exposure to household tobacco smoke	Never	1.00*		1.00*	
	Less than monthly	1.21	1.01–1.46^‡^	1.51	1.25–1.83^‡^
	Monthly	1.11	0.86–1.44	1.06	0.81–1.39
	Weekly	1.23	0.89–1.72	1.17	0.83–1.65
	Daily	1.01	0.80–1.29	0.99	0.77–1.28
Parity (n)	0	1.00*		1.00*	
	1	1.30	1.12–1.51^‡^	1.49	1.28–1.74^‡^
	2	1.68	1.29–2.21^‡^	2.30	1.75–3.04^‡^
	3+	2.10	1.18–3.75^‡^	4.49	2.52–7.98^‡^
Trimester	1^st^	1.00*		1.00*	
	2^nd^	0.85	0.73–0.99^‡^	0.82	0.70–0.96^‡^
	3^rd^	1.00	0.84–1.20	0.69	0.57–0.84^‡^
Prenatal iron and folate supplements	Yes	1.00*		1.00*	
	No	1.06	0.66–1.70	1.06	0.65–1.71
				

RRRs for a given risk factor are adjusted for all other tabulated risk factors. Mild anemia and moderate-to-severe anemia are defined as 10 g/dl ≤ hemoglobin (Hb) < 11 g/dl and Hb < 10 g/dl, respectively.

* Baseline category.

^‡^ Differs significantly from 1 at the 95% confidence level.

Abbreviations: RRR = relative risk ratio, 95% CI = 95% confidence interval, BMI = body mass index.

With the exception of prenatal iron and folate supplementation, all the other predictor variables had statistically significant effects on the relative risks of mild and moderate-to-severe anemia in pregnancy. For example, the relative risk of moderate-to-severe anemia in pregnancy was 0.30 (95% CI = 0.17–0.57) times smaller for women with a university or college education than for women with no formal schooling.

## Discussion

Our study is the first, to our knowledge, to examine the impact of biomass smoke on anemia in pregnant women. Consistent with our hypotheses, our analysis showed that—in the rural setting of Nagpur, India—the prevalence of any anemia in pregnancy and of moderate-to-severe anemia in pregnancy was higher among women who live in households using biomass fuels than among women who live in households using clean fuels. In addition, we found that exposure to biomass smoke was associated with higher risks of mild and moderate-to-severe anemia in pregnancy, independent of maternal age, BMI, education level, exposure to household tobacco smoke, parity, trimester when hemoglobin was measured, and receipt of prenatal iron and folate supplements.

Our results suggest that household use of biomass fuel for cooking may increase the risk of anemia in pregnant women, independently of other factors. This result is biologically plausible because of the potential role of biomass smoke in triggering systemic inflammation. Systemic inflammation is a well-known cause of anemia, mediated by inflammatory cytokines such as tumor necrosis factor alpha (TNF-α), interleukin-1 (IL-1), interleukin-6 (IL-6), and interferon-γ (IFN-γ). Mechanisms by which these cytokines cause anemia include dysregulation of iron homeostasis, impaired erythropoietin response to reduced hemoglobin levels, and impaired marrow response to erythropoietin [13].

Biomass smoke contains a variety of pollutants that may induce systemic inflammation, including carbon monoxide, transitional metals, ultrafine particles (UFP, < 0.1 μm in diameter), particulate matter of less than 2.5 μm in diameter (PM_2.5_), and particulate matter of less than 10 μm in diameter (PM_10_) [24]. A variety of studies have shown positive associations between one of these components, or biomass smoke as a whole, with serum levels of markers of systemic inflammation. Of particular relevance to our study, work by Dutta, Ray, and Banerjee showed that rural Indian women who cook with biomass fuel have higher serum levels of IL-6, CRP, and TNF-α than those who cook with LPG. Levels of these cytokines were positively associated with levels of PM_2.5_ and PM_10_ in indoor air [24]. In addition, observational studies have shown positive correlations between levels of outdoor exposure to UFP, PM_2.5_, and/or PM_10_ with serum levels of CRP, IL-6, TNF-α, and interleukin-1 (IL-1) [25–27]. Moreover, an interventional study found that controlled exposure to wood smoke particles—wood being by far the most common biomass fuel used by participants in our study—is linked with increased levels of neutrophils, which are involved in the inflammatory process [28].

Three published studies have looked at the impact of biomass smoke on anemia in children, all consisting of secondary analyses of national health survey data: from India by Mishra and Retherford in 2006; from 29 developing countries by Kyu, Georgiades, and Boyle in 2010; and from Swaziland by Machisa, Wichmann, and Nyasulu in 2013. Our findings are consistent with those of Mishra and Retherford, which showed a significant increase in the relative risk of moderate-to-severe anemia in Indian children whose households used biomass fuel compared to those whose households used cleaner fuels (RRR = 1.58, 95% CI = 1.28–1.94) [15]. Kyu, Georgiades, and Boyle reported an association, after adjusting for covariates, between exposure to biomass smoke at the country level and moderate-to-severe anemia in children. They also found an association between exposure to biomass smoke at home with mild anemia but not with moderate-to-severe anemia [19], whereas we found both. In contrast with ours and the other two studies, the study by Machisa, Wichmann, and Nyasulu found no association of biomass fuel use with either mild or moderate-to-severe anemia [29]. In comparing our study with these others, however, it must be remembered that they are in different demographics, namely pregnant women vs. children.

### Strengths and limitations

Strengths of our study include the large sample size and the high participation rate in the target study population. The MNH Registry enrolls nearly all pregnant women in the Nagpur clusters, so our study sample was well representative of this demographic. Another strength of our study is that data were collected by community members who could, for the most part, corroborate survey responses to questions such as fuel type and presence of a smoker in the household.

Our study has various limitations, several of which relate to fuel type. First, fuel type data were limited to the primary fuel type, whereas it is common amongst the study population to use multiple fuel types, including both biomass fuel and clean fuel. Second, we limited our analysis to the fuel type used in the primary house, while 5.3% of study participants spent more than 30 days during their pregnancy in a different house using a different fuel type (clean fuel vs. biofuel). Therefore, our exposure data were not as precise as we would have liked. Third, grouping fuel types into only two categories (biomass fuel and clean fuel) may have obscured the effects of different fuel types on anemia. For example, some of the fuels classified as biomass fuel in this study may have a greater impact on anemia than others.

Another limitation was accuracy of the trimester variable, since it depended on an estimate of gestational age on a date that only approximated the date of hemoglobin measurement. However, since we adjusted for this variable and the distributions across trimesters were essentially equivalent for biomass fuel users and clean fuel users, our inability to more accurately correct for trimester should not have biased the results.

In addition to these limitations in studied variables, other limitations stemmed from unmeasured variables: because this study was a secondary data analysis, it was only possible to control for risk factors of anemia in pregnancy included in the source study. One variable that we were unable to include was inter-pregnancy interval; however, this variable is not quite as relevant in Nagpur as in other low-resource settings because, in our study population, the average parity was relatively low (see Table 1).

Likely the most important confounder in the association of biomass fuel use with anemia in pregnancy is socioeconomic status. Biomass fuel is less expensive than clean fuels, and anemia in pregnancy tracks with poverty. For example, the prevalence of anemia in pregnancy varies with standard of living on a national level: 52% of pregnant women in non-industrialized countries are anemic compared to 23% in industrialized countries [1]. The only index of socioeconomic status for which data were available in this study was education level. While education level is widely used as a metric of socioeconomic status—including in studies of risk factors for anemia in pregnancy [9–11, 30]—correcting for this factor alone means that residual confounding is likely.

Some other potential confounders that we were unable to incorporate in our analysis were iron, vitamin B_12_, and folate levels. While this information would be useful for the purpose of our study, measurement of these levels is not part of the WHO’s recommendations on anemia in pregnancy, as these tests are expensive and have limited utility in resource-poor settings. The WHO recommends that all pregnant women receive daily iron and folate supplementation, with anemic women receiving a double dose of iron [1,31]. The health centers in Nagpur follow these guidelines.

We also could not account for the presence of chronic diseases, parasites, and poor diet or for a measure of general nutritional status, as this information is not available in rural settings such as those in Nagpur. The healthcare facilities in these settings are not equipped for the more detailed assessment of pregnant women that diagnosis of chronic diseases and parasites would require. In terms of diet, it varies over time in rural settings—for example, with the season and food availability; therefore, any assessment of diet during pregnancy would be difficult to perform and would not satisfactorily address diet as a risk factor for anemia.

### Conclusion

Despite these limitations, our study has value as the first to examine the association between biomass fuel use and anemia in pregnancy. This association is biologically plausible as a result of the effect of biomass smoke in triggering systemic inflammation. Moreover, it has major public health implications: anemia in pregnancy is linked with adverse pregnancy outcomes, and interventions to reduce its burden solely through iron supplementation have been met with little success. Given the high prevalence of anemia in pregnancy and the widespread use of biomass fuel for cooking in India and other developing countries, there is an urgent need to both reduce exposure to smoke from biomass fuel and to diagnose and treat anemia in pregnancy.

## Supporting Information

S1 Appendix Interpretation of the relative risk ratio (RRR). This primer on interpretation of RRR makes use of an example provided in S1 Table.
(DOCX)

S1 Table Example of relative risks and relative risk ratios. This example of relative risk ratios is explained in S1 Appendix.
(DOCX)
