# Supplementary material for: Does Smoke from Biomass Fuel Contribute to Anemia in Pregnant Women in Nagpur, India? A Cross-Sectional Study
Source: PLoS One. 2015 May 29;10(5):e0127890. doi: 10.1371/journal.pone.0127890 (PMC4449186; doi:10.1371/journal.pone.0127890)
Supplement: S1 Appendix — This primer on interpretation of RRR makes use of an example provided in S1 Table. (DOCX) [file pone.0127890.s001.docx]

**Interpretation of the relative risk ratio (RRR)**

To understand how to interpret RRR, it may be helpful to go through an example [23]. In this case, our outcomes will be no disease (base outcome), mild disease, and severe disease. Our predictor variable will be exposure to a toxin. For probabilities of each outcome, refer to S1 Table. The relative risk (RR) for a given outcome *i* if exposed to the toxin is calculated as follows, where the notation P(outcome *i* ***|*** exposed) signifies the probability of outcome *i* if exposed:

$\text{RR = }\frac{\text{P(outcome }i\text{|exposed)}}{\text{P(base outcome|exposed)}}$ (1)

For example, the RR for mild disease if exposed is the following:

$\text{RR = }\frac{\text{P(mild disease|exposed)}}{\text{P(no disease|exposed)}}=\frac{0.5}{0.3}\text{=1.67}$ (2)

The RRR for a given outcome *i* then is the ratio of the RR of outcome *i* if exposed and the RR of outcome *i* if unexposed:

$\text{RRR = }\frac{\text{P(outcome }i\text{|exposed)/P(base outcome|exposed)}}{\text{P(outcome }i\text{|unexposed)/P(base outcome|unexposed)}}$ (3)

In the case of mild disease, the RRR is the following:

$\text{RRR = }\frac{\text{P(mild disease|exposed)/P(no disease|exposed)}}{\text{P(mild disease|unexposed)/P(no disease|unexposed)}}=\frac{1.67}{0.5}=3.33$ (4)

The RRR of 3.33 signifies that the RR of mild disease (compared to no disease) increases 3.33 times with exposure to the toxin.
