# Supplementary material for: Does Smoke from Biomass Fuel Contribute to Anemia in Pregnant Women in Nagpur, India? A Cross-Sectional Study
Source: PLoS One. 2015 May 29;10(5):e0127890. doi: 10.1371/journal.pone.0127890 (PMC4449186; doi:10.1371/journal.pone.0127890)
Supplement: S1 Table — This example of relative risk ratios is explained in S1 Appendix. (DOCX) [file pone.0127890.s002.docx]

**S1 Table. Example of relative risks and relative risk ratios**

| **Outcome *i*** | **P(outcome *i* \| unexposed)** | **P(outcome *i* \| exposed)** | **RR in unexposed** | **RR in exposed** | **RRR** |
| --- | --- | --- | --- | --- | --- |
| No disease (base) | 0.6 | 0.3 | 1 | 1 | 1 |
| Mild disease | 0.3 | 0.5 | 0.5 | 1.67 | 3.33 |
| Severe disease | 0.1 | 0.2 | 0.167 | 0.67 | 4 |

P(outcome *i* ***|*** unexposed) signifies the probability of outcome *i* if unexposed. All relative risks and relative risk ratios are calculated using the outcome of no disease as the base outcome.

Abbreviations: RR = relative risk, RRR = relative risk ratio.
